# Supplementary material for: WordCommentsAnalyzer: A windows software tool for qualitative research
Source: F1000Res. 2018 Sep 4;7:536. Originally published 2018 May 3. [Version 2] doi: 10.12688/f1000research.14819.2 (PMC6137411; doi:10.12688/f1000research.14819.2)
Supplement: Supplementary file 3 [file f1000research-7-17614-s0002.tgz › b2a79496-4ada-47c4-9682-a99ce867836d.zip!/Eight Iranian Health Journals/Yafteh _32.docx]

© Yafteh  . This work is licensed under CC BY 4.0 (https://creativecommons.org/licenses/by/4.0/).

Volume & Issue: دوره 12 - شماره 4

Title: بررسی میزان ماستیت بالینی و تحت بالینی بر اساس خصوصیات شیمیایی و فیزیکی شیر مادر

http://yafte.lums.ac.ir/article-1-325-fa.html

ماستیت یا التهاب پستان عارضه‌ای است که در حدود 10% از مادران شیرده مشاهده می شود و شایع‌ترین ضایعه‌ی التهابی پستان در مادران شیرده است . در این عارضه شیر مادر دچار تغییرات شیمیایی و فیزیکی می گردد . این مسئله می تواند منجر به افت شیردهی مادر، کاهش وزن نوزاد و در نتیجه اختلال رشد وی گردد.این تحقیق با هدف تعیین میزان ماستیت بالینی و تحت بالینی بر اساس خصوصیات شیمیایی و فیزیکی شیر مادران صورت گرفت. بحث و نتیجه گیری: نتایج این مطالعه نشان می دهد که ماستیت برروی ترکیبات شیمیایی و خصوصیات فیزیکی شیر مادر تاثیر‌گذار است؛ به طوری‌که میزان چربی،پروتئین و قند شیر مادران مبتلا به ماستیت نسبت به شیر مادران سالم کاهش می‌یابد؛ همچنین pH ، دانسیته و نقطه‌ی انجماد شیر مادران مبتلا نسبت به مادران سالم کاهش نشان می‌دهد . یافته ها: بر اساس نتایج به دست آمده در این پژوهش ، 13 نمونه ( 7/5% مادران) مبتلا به ماستیت بودند . نتیجه‌ی کشت نمونه‌های شیر مادران مبتلا به ماستیت در 12 نمونه ( 6/99% ) رشد استافیلوکوک‌های کواگولاز منفی و در یک نمونه ( 4/0% ) رشد اشیریشیا کلی را نشان داد . بین میانه‌ی مقدار قند، پروتئین، pH و دانسیته‌ی شیر در دو گروه، تفاوت معنی داری وجود دارد مواد و روش ها: مطالعه‌ی حاضر از نوع توصیفی مقطعی می باشد. جامعه مورد مطالعه، 229 مادر شیرده مراجعه کننده به مراکز بهداشتی درمانی شهر خرم آباد بودند که به طور خوشه‌ای تصادفی انتخاب شدند و نمونه‌ی شیر آنها جمع آوری گردید. ابزار گردآوری داده‌ها پرسشنامه بود. اطلاعات کسب شده در نرم افزار آماری SPSS ثبت گردید و جهت ارتباط سنجی از آزمون‌های آماری کای اسکوار، من ویتنی و T-testاستفاده شد. نتایج در سطح معنی داری 5% گزارش شده است.

Title: اندازه گیری مقدار فلزات سرب وکادمیم نمونه های برنج پر مصرف استان لرستان و مقایسه آن با استاندارد های ملی

http://yafte.lums.ac.ir/article-1-326-fa.html

برنج یکی از اجزاء اصلی سبد غذایی مردم کشور و حدود 4/2 بیلیون نفر از جمعیت جهان است، اگر چه از نظر سطح زیر کشت پس از گندم قرار دارد ولی 85% از کل تولید آن به مصرف تغذیه انسان می رسد. در ایران سرانه مصرف برنج 5/42 کیلوگرم برآورد می شود که در واقع دومین محصول پرمصرف کشور است. هدف از این مطالعه تعیین میزان فلزات سنگین وسمی در برنج های پرمصرف کشت شده در نواحی مختلف استان لرستان بود.مواد و روش ها: دراین مطالعه 99 نمونه برنج طارم و دمسیاه کشت شده در سه ناحیه استان لرستان جمع آوری شدومقادیر سرب و کادمیم بااستفاده از دستگاه اسپکتروفتومتر جذب اتمی مدل BRAIC WFX-130 تعیین گردید.پس از جمع آوری اطلاعات با استفاده از نرم افزار آماری SPSS نتایج با استاندارد های ملی مقایسه شد. بحث و نتیجه گیری: مقادیر سرب و کادمیوم در نمونه های برنج کشت شده در استان لرستان کمتر از حد مجاز می باشد واز این نظر مشکلی برای سلامت انسان وجود ندارد. یافته ها: مقدار کادمیوم و سرب به ترتیب 06/0±037/0، و08/0±077/0 میلی گرم در کیلوگرم ماده غذایی بدست آمد.

Title: بررسی عوامل موثر بر خارش در بیماران مبتلا به نارسایی پیشرفته کلیه تحت درمان با همودیالیز در بیمارستان ولیعصر اراک

http://yafte.lums.ac.ir/article-1-327-fa.html

خارش یکی از آزاردهنده ترین علائم در بیماران مبتلا به نارسایی مزمن کلیه است که در بسیاری از آنها دیده می شود ،خارش اثرات منفی بر کیفیت زندگی بیمار می گذارد به طوری که هم برای بیمار و هم پزشک بسیار مشکل ساز است . پاتوژنژ دقیق آن هنوز مشخص نشده است . هدف از انجام این مطالعه تعیین عوامل دخیل در خارش در این بیماران می باشد بحث و نتیجه گیری: خارش هنوز یک مسئله شایع در بیماران مبتلا به نارسایی مزمن کلیه تحت همودیالیز است.میزان بالای فسفر سرم فاکتور مهمی در ایجاد خارش در این بیماران بنظر می رسد. یافته ها: از میان بیماران دیالیزی48% زن و 52% آنان مرد بودند. علت عمده نارسایی کلیه در آنها، پرفشاری خون، کلیه پلی کیستیک، گلومرولونفریت، مسائل اورولوژی، دیابت و... بود . 45 % بیماران از خارش شاکی بودند. بین شدت خارش و جنس ، بیماری زمینه ای ، طول مدت دیالیز ، سطح هموگلوبولین ،آلبومین ، پاراتورمون ، آلکالن فسفاتازکلسیم و کراتینین سرم ارتباط معنی داری وجود نداشت. اما بین شدت خارش با سن افراد و سطح فسفر ارتباط مستقیم وجود داشت. مواد و روش ها: این مطالعه توصیفی مقطعی بر روی 100 بیمار دیالیزی در مرکز دیالیز بیمارستان ولیعصر اراک انجام شد . شدت خارش بر حسب شاخص VAS (Visual analogue Scale ) ارزیابی شد و در همان زمان سطح کلسیم ، فسفر ، آلبومین ، هموگلوبولین، اوره، کراتینین، آلکالن فسفاتاز، پاراتورمون سرم اندازه گیری شده پس از جمع آوری اطلاعات ، داده ها با استفاده از آزمون های آماری تجزیه و تحلیل گردید.

Title: بررسی تاثیر امگا 3 و فلووکسامین در بهبودی بیماران دو قطبی نوع اول مراجعه کننده به

http://yafte.lums.ac.ir/article-1-328-fa.html

اختلال دو قطبی نوع اول یکی از ناتوان کننده ترین سندرمهای روانپزشکی است. درمان این اختلال بطور عمده استفاده از داروهای تثبیت کننده خلق، مداخلات روانی اجتماعی و در موارد شدید استفاده از تشنج برقی است . از آنجا که در سالهای اخیر توجه خاصی به منابع سرشار از امگا 3 در درمان بسیاری از امراض به ویژه بیماریهای روانی شده است بر آن شدیم تاثیر درمانی امگا3 در مقایسه با فلو وکسامین را در درمان فاز افسردگی بیماران دو قطبی مراجعه کننده به درمانگاه تخصصی اعصاب و روان انجام دهیم. بحث و نتیجه گیری: با توجه به یافته های تحقیق مبنی بر تاثیر مکمل امگا3 و با عنایت به بی ضرر بودن استفاده از این دارو پیشنهاد می گردد از امگا3 به عنوان درمان کمکی در کنار سایر داروهای ضد افسردگی بیماران استفاده گردد یافته ها: نتایج تحقیق نشان داد که میانگین نمره افسردگی هامیلتون در هر دو گروه تحت مطالعه در بدو مطالعه و هفته های2 ، 4 ، 8 و 12 به میزان قابل توجهی کاهش یافته و میزان عملکرد بیماران بهبود یافته است . آزمون آماری Repeted measures تفاوت معنی داری در میزان نمره افسردگی دو گروه قبل از مداخله درمانی ، هفته های 2 ، 4 ،8 و 12 ( ماههای اول ، دوم و سوم ) بعد از شروع درمان نشان داد.مواد و روش ها: مطالعه حاضر یک کارآزمائی بالینی است. در این مطالعه 80 بیمار بطور کاملا تصادفی دو سو کور در دو گروه مورد ( تحت درمان با فلووکسامین وکپسول امگا 3 ) و شاهد ( تحت درمان با فلووکسامین) قرار گرفتند . کلیه بیماران در بدو ورود به مطالعه و هفته های 2، 4 ، 8 و 12 از طریق تست افسردگی هامیلتون و مصاحبه بالینی روانپزشک مورد بررسی قرار گرفتند . اطلاعات پس از جمع آوری مورد تجزیه و تحلیل آماری قرار گرفت.

Title: بررسی فراوانی گرانولوم جسم خارجی در ریه اجساد سوء مصرف کنندگان مواد تزریقی ، ارجاع شده به سالن تشریح پزشکی قانونی تهران در سال 1387

http://yafte.lums.ac.ir/article-1-329-fa.html

تشخیص پس از مرگ سوء مصرف مواد برای تعیین علت فوت و بررسی عوامل موثر در حوادث منجر به فوت اهمیت ویژه ای دارد. یکی از روشهای تشخیصی، بررسی آسیب شناسی ریه از نظر وجود گرانولوم جسم خارجی است. مواد و روش ها: مطالعه بصورت تحلیلی مقطعی در سالن تشریح پزشکی قانونی تهران روی اجساد دارای علایم خطر انجام شد. در مواردی که آزمایش سم شناسی ادرار یا صفرا از نظر مواد مخدر مثبت شد نمونه بافت ریه تحت آزمایش آسیب شناسی قرار گرفت. بحث و نتیجه گیری: در تعداد قابل توجهی از نمونه های آسیب شناسی ریه معتادین تزریقی گرانولوم جسم خارجی مشاهده می گردد. می توان بعنوان روشی قابل اعتماد برای تشخیص اعتیاد تزریقی از آن استفاده نمود یافته ها: در مجموع 90 جسد مورد بررسی قرار گرفتند. میانگین سنی قربانیان 05/33 سال با انحراف معیار 45/9 سال بود . دامنه سنی از 17 تا 60 سال و 8/97 درصد قربانیان مذکر بودند. نوع ماده مورد استفاده در 53 مورد بر اساس شرح حال مشخص بود که در میان آنها 38/40% قربانیان کراک، 15/21% هروئین، 92/26% تریاک، 69/7% شیشه، 84/3% قرص و 61/9% همگی انواع مواد مخدر را مصرف نموده بودند. 2/72% موارد صرفاً یک ماده و 8/27% قربانیان بیش از یک دارو مصرف نموده بودند. نمونه های ریه 1/91% اجساد از نظر وجود جسم خارجی ریه مثبت بودند. بین سن و جنس قربانی و وجود جسم خارجی در ریه ارتباط معنی داری وجود نداشت.

Title: تحریک تخمک گذاری ، اندومتر و لانه گزینی

http://yafte.lums.ac.ir/article-1-330-fa.html

کلیات موضوع: در این مقاله مجموعه مطالعات انجام گرفته در زمینه تاثیر تحریک تخمک گذاری بر اندومتر رحم و تاثیر آن بر لانه گزینی مورد بررسی قرار گرفته است. بحث و نتیجه گیری: بررسی های محققین نشان داده است که تحریک تخمک گذاری باعث تغییرات نامطلوبی در اندومتر رحم می شود که این تغییرات باعث نقص در اتصال جنین به آندومتر و در نهایت درصد پائین لانه گزینی جنین می شود. با توجه به تحقیقات انجام شده و اهمیت استفاده از روش تحریک تخمک گذاری در درمان نازایی و از طرفی تاثیرات نامطلوبی که تحریک تخمک گذاری بر روی اندومتر رحم در زمان لانه گزینی جنین دارد در مجموع ضروری به نظر می رسد به منظور بهبود روشهای درمانی در کلینیکهای ناباروری تحقیقات بیشتری مورد نیاز استتاریخچه: گروه مناش برای اولین بار از روش تحریک تخمک گذاری در کلینیکهای ناباروری استفاده کردند و میزان حاملگی را با استفاده از این روش افزایش دادند. اما به علت برهم خوردن تعادل هورمونها در استفاده از این روش و تاثیر این تغییرات هورمونی بر اندومتر رحم، درصد موفقیت لانه گزینی جنین کاهش می یافت.

Title: مقایسه دریافت مواد غذایی مؤثر بر علایم بالینی آرتریت روماتوئید : مطالعه مورد- شاهدی

http://yafte.lums.ac.ir/article-1-331-fa.html

آرتریت روماتوئید (RA) یکی از بیماریهای مزمن شایع است. برخی سبزیها و روغنها می توانند التهاب را کاهش دهند و احتمالا مرکبات، گوجه فرنگی، گوشت قرمز و ترشیجات شور درد و سفتی مفاصل را تشدید کنند. هدف این مطالعه، مقایسه دریافت رژیمی برخی غذاها در بیماران زن مبتلا به آرتریت روماتوئید در مقایسه با گروه کنترل می باشد. مواد و روش ها: در این مطالعه مورد- شاهدی، 44 زن مبتلا به RA با 41 زن سالم جور شده از نظر سن، مقایسه شدند. پرسشنامه کمی بسامد غذایی به شکل مصاحبه برای دستیابی به الگوی غذایی پر شد. داده ها با آزمون کولموگروف اسمیرنوف و هیستوگرام برای تست نرمال بودن توزیع داده ها و آزمون من یو ویتنی برای تعیین اختلاف بین دو گروه تجزیه و تحلیل گردید. یافته ها: میانگین± انحراف معیار نمایه توده بدنی (BMI) در گروه های بیمار ((RA و کنترل به ترتیب برابر با 95/3±86/28 و 32/4±23/28 بود. دریافت هفتگی گوجه فرنگی، طالبی، چای و اسفناج در گروه مورد بیشتر از شاهد بود (001/0 >p)؛ گرچه ماست(001/0 >p)، توتها(01/0 >p)، هلو(001/0 >p)، قهوه(001/0 >p)، غذاهای آماده(009/0 =p)، سرخ کرده ها(003/0=p) و انبه (001/0 >p) غذاهای با مصرف کمتر در گروه RA نسبت به گروه کنترل بودند. بحث و نتیجه گیری: بیماران RA میزان اضافه وزن بالاتری داشتند، اما تفاوت معنی داری بین دو گروه وجود نداشت. دریافت برخی غذاهای آلرژی زا مانند گوجه فرنگی و طالبی در گروه RA بالاتر بود، در حالی که برخی غذاهای آنتی اکسیدان در بیماران RA کمتراز گروه کنترل بود، به همین علت، کاهش وزن و مصرف بیشتر سبزیجات تازه توصیه می شود.

Title: بررسی سابقه بیماری های ایسکمیک قلبی در والدین کودکان مبتلا به کاوازاکی و ارتباط آن با شدت عوارض و عود بیماری در کودک

http://yafte.lums.ac.ir/article-1-332-fa.html

بیماری کاوازاکی (Kawasaki Disease یا سندروم جلدی ـ مخاطی غدد لنفاوی) یک واسکولیت سیستمیک شایع در کودکان است . علیرغم ناشناخته ماندن علت بیماری؛ عوامل عفونی، ژنتیکی و پاسخ ایمنی در ایجاد بیماری کاوازاکی مؤثر دانسته شده اند. عوارضی چون آنوریسم شریان کرونر، کاردیومیوپاتی و نارسایی قلبی ممکن است رخ دهد که می تواند منجر به ناتوانی و مرگ و میر قابل ملاحظه در کودکان گردد. این بیماری علت اصلی بیماری اکتسابی قلبی در کودکان ژاپنی و امریکایی بوده است. بحث و نتیجه گیری: بین میزان بروز پرولاپس میترال در مادران و بیماری ایسکمیک قلبی در پدران بیماران مبتلا به کاوازاکی و شدت بیماری در فرزندان آنها ارتباط معنی دار وجود دارد. هم چنین با بررسی اندکس هایی مانند اکوکاردیوگرافی، آنزیم های کبدی،ESR ، CRP و میزان سدیم می توان فرم شدید و خفیف را از یکدیگر تشخیص داد. برای به دست آمدن نتایج جامع تر نیاز به مطالعات مقایسه ای آینده نگر و چندمرکزی و با حجم نمونه بیشتراست. یافته ها: 50 بیمار (32 پسر و 18 دختر) با میانگین سنی 43 ماه وارد مطالعه شدند. همه بیماران تب دار بودند و آنوریسم شریان کرونر در 2% آنها دیده شد. عود در هیچ بیماری مشاهده نشد و سابقه کاوازاکی در والدین و خواهران و برادران کودکان نیز منفی بود. پرولاپس میترال در مادران و هم چنین بیماری ایسکمیک قلبی در پدران کودکانی که بیماری شان شدید بود به طور معنی داری از گروه خفیف بالاتر بود (به ترتیب03/0 p= و001/0 p=). هم چنین رگورژیتاسیون میترال و تعداد دفعات گرفتن ایمونوگلوبولین وریدی در گروه شدید به طور معنی دار از گروه خفیف بالاتر بود (به ترتیب02/0 p= و008/0 p=). مواد و روش ها: در این مطالعه مقطعی به بررسی پرونده 61 کودک مبتلا به کاوازاکی اثبات شده که از دی ماه سال 1383 تا بهمن ماه سال 1386 در بیمارستان مفید بستری شده بودند و همچنین والدین آنها پرداختیم. پس از حذف بیمارانی که معیارهای خروج از مطالعه را داشتند، 50 بیمار به دو دسته شدید و خفیف تقسیم شدند. داده های به دست آمده توسط ویرایش 5/11 نرم افزار SPSS مورد آنالیز قرار گرفت. 05/0p< معنی دار درنظر گرفته شد.

Title: بررسی اپیدمیولوژیک بیماری ترومای تجمعی در کارمندان دستگاههای دولتی شهر کرمان در سال 1386

http://yafte.lums.ac.ir/article-1-333-fa.html

به علت وجود عوامل مخاطره آمیز گوناگون در محیط کار امکان بروز بیماریهای شغلی امری ثابت شده است . بیماری ترومای تجمعی یا آسیبهای مکرر در واقع بیانگر صدماتی است که در اثر حرکات فیزیکی و تکراری و فشارهای هر چند جزئی به عضلات و تاندونها و بقیه بافتهای نرم بدن وارد می شود . این بیماری شایع ترین بیماری ناشی از کار نیز شناخته شده است. مواد و روش ها: در این تحقیق نوع مطالعه توصیفی و از نوع مقطعی و جمعیت مورد مطالعه ، کارمندان دستگاههای دولتی شهر کرمان در سال 1386 بود. داده‌های تحقیق با استفاده از فرم معاینه و 3 پرسشنامه‌ سنجش بیماری‌های مرتبط با کار، رضایت شغلی، استرس شغلی در بین کارمندان ادارت دولتی کرمان جمع‌آوری گردیده است. پس از تکمیل پرسشنامه ها توسط پژوهشگر آموزش دیده اطلاعات جمع آوری شده ، جمع بندی و توسط نرم افزار SPSS 13و با استفاده از آزمون آماری کای دو آنالیز شد. مقادیر p کوچکتر یا مساوی 05/0 معنی دار در نظر گرفته شد. یافته ها: تحلیل یافته ها نشان داد که مواردی چون تکرار نوشتن ، تایپ کردن ، کار با کامپیوتر در محیط کار، تکرار بلند کردن اجسام در حد روی میز ، تکرار تردد در طبقات از طریق پله ، تکرار تردد در اداره از طریق راهرو ، رسیدن کف پا به زمین هنگام نشتن روی صندلی، استفاده از کفی شیب‌دار زیر پا، وضعیت صندلی با پرکنندگی کمر، وضعیت صندلی با پشتی بلند تا حد کتف، وضعیت صندلی با پایه‌های چرخان علت ایجاد کننده بیماری ترومای تجمعی نبوده است. اماخم و راست شدن و تکیه ندادن به صندلی هنگام نشستن در ایجاد بیماری موثر بودند. از جمله یافته های دیگر تحقیق وضعیت آگاهی ارگونومیکی، رضایت شغلی و استرس شغلی در کارمندان بود که با توجه به میانه نمره محاسبه شده برای هر سه مورد متوسط ارزیابی شد. بحث و نتیجه گیری: انجام حرکات تکراری به صورت خم و راست شدن و عدم تکیه دادن به صندلی در هنگام نشستن عوامل مؤثر و معنی داری در شیوع بالای سندرم ترومای تجمعی در کارمندان شهر کرمان بودند. تعدیل این حرکات و یا حتی استفاده از یک حمایت کننده کمری ممکن است بتواند از این شیوع بالای بیماری بکاهد. کمترین شیوع بیماری ترومای تجمعی در کارمندان زیر 30 سال مشاهده گردید که این میزان با افزایش سن و سنوات کار به صورت خطی افزایش می یافت.
